# Identification of Isoform-Selective Ligands for the Middle Domain of Heat Shock Protein 90 (Hsp90)

**DOI:** 10.3390/ijms20215333

**Published:** 2019-10-26

**Authors:** Oi Wei Mak, Raina Chand, Jóhannes Reynisson, Ivanhoe K. H. Leung

**Affiliations:** 1 School of Chemical Sciences, The University of Auckland, Private Bag 92019, Victoria Street West, Auckland 1142, New Zealand; omak089@aucklanduni.ac.nz (O.W.M.); rcha387@aucklanduni.ac.nz (R.C.); 2 School of Pharmacy and Bioengineering, Hornbeam Building, Keele University, Keele, Staffordshire ST5 5BG, UK; 3 Maurice Wilkins Centre for Molecular Biodiscovery, The University of Auckland, Private Bag 92019, Victoria Street West, Auckland 1142, New Zealand

**Keywords:** Hsp90, virtual screening, intrinsic tryptophan fluorescence, ligand binding, isoform-selective

## Abstract

The molecular chaperone heat shock protein 90 (Hsp90) is a current inhibition target for the treatment of diseases, including cancer. In humans, there are two major cytosolic isoforms of Hsp90 (Hsp90α and Hsp90β). Hsp90α is inducible and Hsp90β is constitutively expressed. Most Hsp90 inhibitors are pan-inhibitors that target both cytosolic isoforms of Hsp90. The development of isoform-selective inhibitors of Hsp90 may enable better clinical outcomes. Herein, by using virtual screening and binding studies, we report our work in the identification and characterisation of novel isoform-selective ligands for the middle domain of Hsp90β. Our results pave the way for further development of isoform-selective Hsp90 inhibitors.

## 1. Introduction

Heat shock protein 90 (Hsp90) is a class of 90 kDa dimeric chaperone proteins from the heat shock protein family [1,2,3,4,5,6,7,8,9,10,11]. Members of the Hsp90 family are highly conserved and can be found in almost all organisms (except for archaea) [12,13,14]. The main function of Hsp90 is to maintain the proper folding of proteins within the cell [1,2,3,4,5,6,7,8,9,10,11]. Its specific tasks include the assistance of folding of the nascent polypeptide chains, the stabilisation of proteins (in particularly during heat shock or other stress conditions), and catalysing the refolding of denatured proteins [1,2,3,4,5,6,7,8,9,10,11]. Due to the central role Hsp90 plays in protein folding and stabilisation, the protein is involved in many important cellular processes. These include (but are not limited to) the regulation of cellular homeostasis, cell cycle control, signal transduction, and cytoprotection [1,2,3,4,5,6,7,8,9,10,11].

Structurally, each Hsp90 monomer comprises three domains [4,6,9,10,11], which are termed the N-terminal domain (NTD), middle domain (MD), and C-terminal domain (CTD). The NTD is joined with the MD through a flexible linker, which is named the charged region. The NTD binds and catalyses the hydrolysis of adenosine triphosphate (ATP), which induces conformational change that is essential to the chaperone function of Hsp90 [15,16]. The CTD contains the homodimerisation site [17,18], whilst the MD is responsible for client protein binding [19,20]. The MD also modulates the ATPase activity of the NTD through interactions with the γ-phosphate moiety of ATP [19,20]. The activity of Hsp90 is mediated by a number of co-chaperones [21,22]. All three domains are involved in the binding of different co-chaperones [21,22].

In humans, there are two major cytosolic isoforms of Hsp90, which are Hsp90α and Hsp90β (Table S1) [23]. There are inherent differences between the cellular processes that the two isoforms are involved in. Hsp90β is constitutively expressed and it is associated with long-term processes such as cellular adaptation [23,24,25,26]. In contrast, Hsp90α is involved in processes that require fast response, presumably because it is inducible and shows different client protein preferences under stress conditions [23,24,25,26,27].

Hsp90 is a current inhibition target for the treatment of diseases including cancer. This can be attributed to Hsp90’s ability to maintain and regulate oncoproteins [28,29], as well as those proteins that aid the initiation and progression of tumours [5,30]. For example, overexpression of Hsp90α is often found in acute and chronic tumours (but not benign tumours), and Hsp90β has been implied in multidrug resistance in chronic tumours [23]. There is, therefore, a great anticipation towards the development of Hsp90 inhibitors with cancer therapeutic potential. For example, the inhibition of Hsp90 has been shown to lead to cell cycle arrest and apoptosis of cancerous cells [31]. The inhibition of Hsp90 may also help overcome resistance in chemotherapy [32,33,34].

Numerous inhibitors of Hsp90 have been discovered in the last 15 years [35,36,37,38,39,40,41,42,43,44,45,46,47]. Most of them target binding sites at the NTD and CTD. For example, geldanamycin [48,49], 17-*N*-allylamino-17-demethoxygeldanamycin [50], and ganetespib (also known as STA-9090) [51,52] target the ATP binding pocket at the NTD, and novobiocin was found to bind the CTD of Hsp90 [53]. In spite of the discovery of numerous inhibitors that target the NTD and CTD, there are only few studies on the inhibition of MD [54,55]. It is also worth noting that all 17 small molecule Hsp90 inhibitors that have entered clinical trials to date target the NTD of both isoforms of Hsp90 [35,36,37,38,39,40,41,42,43,44,45,46,47]. Only a handful of isoform-selective Hsp90 inhibitors have been reported [55,56,57,58,59,60,61,62].

We are interested in the development of novel Hsp90 inhibitors. In particular, the isoform-selective Hsp90 inhibitors are of importance because they may give better clinical outcomes than pan-Hsp90 inhibitors [63,64,65]. In addition, isoform-selective inhibitors will also enable studies on the roles of the different Hsp90 isoforms without resorting to genetic manipulations, which are often more invasive. The MD of Hsp90 is of particular interest because the possibility exists to develop isoform-specific Hsp90 binders [55,56] due to the inherit differences in client protein selectivity of the two isoforms especially during stress conditions [27]. Herein, we report our work in the application of virtual high-throughput screening and binding experiments, a strategy that we have previously used to discover Hsp90 NTD inhibitors [66], to identify novel isoform-selective ligands for MD of Hsp90β. Our report provides novel scaffolds for future development of isoform-selective Hsp90β MD inhibitors.

## 2. Results and Discussion

We first investigated potential binding pockets on the MD of Hsp90β that could be targeted by small-molecule ligands. Unlike the NTD and CTD of Hsp90, the MD does not have a well-defined binding site for small molecule ligands. We therefore performed blind docking experiments to predict the most probable binding site for small molecule ligands [67,68]. Gambogic acid, an isoform-selective inhibitor of the MD of Hsp90β [55], was used as the model ligand, and the crystal structure of the MD of human Hsp90β (PDB ID: 3PRY, resolution 2.28 Å) was used as the target. Eighty-seven different potential binding sites on Hsp90β were evaluated by using a consensus scoring approach [69,70] that is based on four different scoring functions, GoldScore (GS) [71], ChemScore (CS) [72,73], piecewise linear potential (ChemPLP) [74], and Astex Statistical Potential (ASP) [75] (Table S2). Our docking results revealed a site within a 10 Å radius centring around the oxygen atom on the sidechain of Asp-367 (x = 8.806, y = 23.993, z = 27.785; Figure S1) as the most probable pocket that could be targeted by small molecules. The binding pocket that we identified is consistent with previous molecular modelling work that suggested the region consisting of residues 350 to 436 as the potential binding site for gambogic acid [55].

High-throughput virtual screening was then conducted against the identified binding site. In total, 9051 molecular entities were downloaded from the natural product library from the InterBioScreen Ltd collection. Ten docking runs were carried out for each ligand with 30% search efficiency. From the consensus scoring, all ligands with low CS (<15.0), GS (<51.0), ChemPLP (<55.0), and ASP (<25.2) scores, as well as those with limited hydrogen bonding (HB < 1) were eliminated in the initial screen. 913 candidates remained, and they were screened again with 100% default search efficiency and 50 genetic algorithm (GA) runs. The candidates with low scores on CS (<20.0), GS (<60.0), ChemPLP (<60.0), ASP (<25.0), and HB (<1) were then filtered out, resulting in 152 compounds. The 152 candidate compounds all have docking scores higher than the score that we obtained by docking gambogic acid to Hsp90β. Visual inspection was then conducted for consensus of the best predicted configuration of the ligands between each scoring function. Ligands that showed implausible configurations, i.e., those that are strained, with lipophilic moieties pointing towards aqueous environment, and those that contain undesirable moieties (e.g., those that are linked to cell toxicity and chemical reactivity), were eliminated.

**Figure 1:**
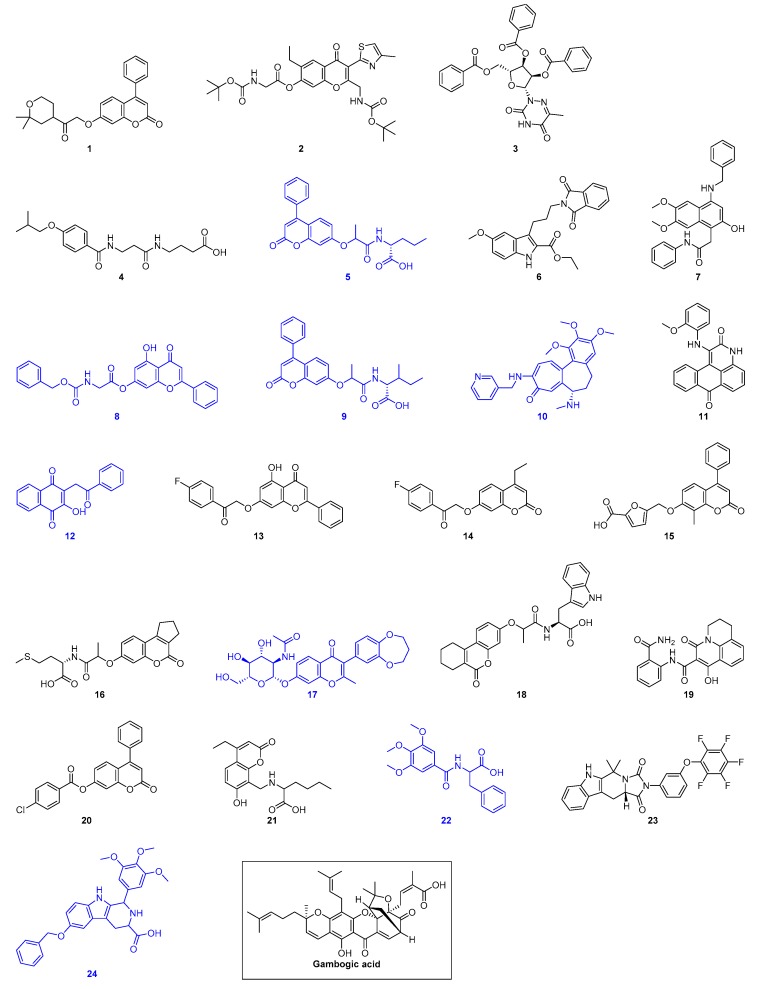
Structures of the 24 virtual hits and gambogic acid (a reported Hsp90 MD inhibitor). Blue indicates compounds that show binding to Hsp90α/β MD as monitored by intrinsic tryptophan fluorescence spectroscopy.

As a result of virtual screening, 24 hit compounds (Figure 1 and Table S3) were selected for further experimental testing against recombinant human Hsp90α MD (residues 294 to 554, Table S4) and human Hsp90β MD (residues 286 to 546, Table S5). As there are tryptophan residues located within the defined binding pocket in both Hsp90α MD and Hsp90β MD, intrinsic tryptophan fluorescence quenching experiment was used to screen the binding of our virtual hits to the proteins. Initial screening experiments were conducted by using 20 μM of Hsp90α/β MD and 1 mM of compounds. Molecules that gave less than 40% fluorescence quenching were eliminated. Our results revealed that eight compounds (**5**, **8**, **9**, **12**, **17**, **22**, **24**) bind to both Hsp90α MD and Hsp90β MD. In addition, one compound (**10**) was found to induce significant quenching (~90%) of Hsp90β MD but not with Hsp90α MD (~20%) (Figure 1, Table S6, and Figure S2).

We then focussed on studying the binding of our compounds (**5**, **8**, **9**, **10**, **12**, **17**, **22**, **24**) to both Hsp90α MD and Hsp90β MD. Titration experiments were performed to quantify the binding affinity (dissociation constant, *K*_D_) to Hsp90α MD and Hsp90β MD (Table 1 and Figures S3–S10). The *K*_D_ values of gambogic acid were also measured as control (Table 1 and Figure S11). Our results show that compounds **5**, **9**, **10**, **12**, **17**, and **24** bind stronger to Hsp90β MD than to Hsp90α MD. The strongest binder was **12**, which binds to Hsp90β MD with a *K*_D_ value of < 20 μM, and a *K*_D_ value of 92 ± 0.8 μM to Hsp90α MD. The seven-fold preference for the β-isoform is similar to gambogic acid, which has a *K*_D_ value of 195 ± 5 μM with Hsp90α MD, and a *K*_D_ value of 33 ± 2 μM with Hsp90β MD. Our *K*_D_ values for gambogic acid are in agreement with previous studies (~10-fold preference) [55]. The selectivity for the β-isoform (over α-isoforms) was progressively less for weaker binders. For example, compound **9**, which bind Hsp90β MD with a *K*_D_ value of 57 ± 1 μM, only shows a 5-fold selectivity for Hsp90β MD. The selectivity of compound **5**, a structure analogue of compound **9** that binds Hsp90β MD with a *K*_D_ of 136 ± 1 μM, was found to be ~3-fold. Compounds **10**, **17,** and **24** (which bind Hsp90β MD with *K*_D_ values > 100 μM) only show ~2- to 3-fold preference for the β-isoform. Compounds **8** and **22**, which bind Hsp90β MD with *K*_D_ > 500 μM, actually have a slight preference for Hsp90α MD, with *K*_D_ values of 284 ± 4 μM and 459 ± 7 μM, respectively.

**Figure 2:**
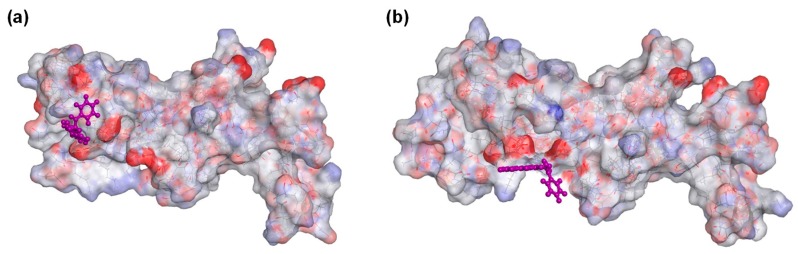
Docking of compound **12** to (**a**) Hsp90α MD (PDB ID: 3Q6M) binding pocket (10 Å around Glu-375) and to (**b**) Hsp90β MD (PDB id: 3PRY) binding pocket (10 Å around Asp-367). For aesthetic reason, residues 359–492 and 350–484 were shown for Hsp90α and Hsp90β, respectively. Both of the displays were processed from the ligand poses as predicted by the GoldScore (GS) scoring function.

**Figure 3:**
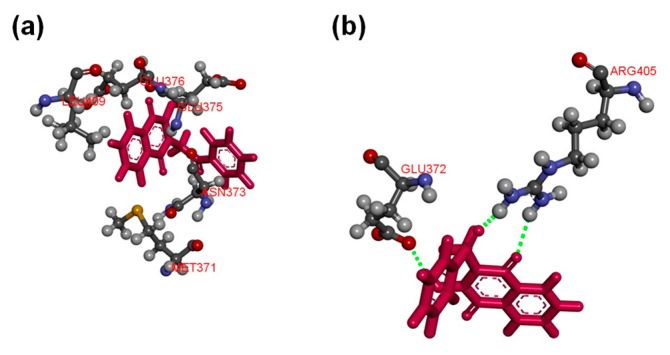
(**a**) Compound **12** does not form any hydrogen bonding interactions with the neighbouring amino acid residues of Hsp90α MD (PDB ID: 3Q6M) at the binding pocket (10 Å around Glu-375); (**b**) compound **12** forms hydrogen bonds with residues Glu-372 and Arg-405 of Hsp90β MD (PDB id: 3PRY) at the binding pocket (10 Å around Asp-367). Both of the displays were processed from the ligand poses as predicted by the GS scoring function.

Molecular docking of compounds **5**, **8**, **9**, **10**, **12**, **17**, **22**, and **24** were then conducted to predict the binding modes and relative energies of the compounds to the two isoforms of Hsp90 MD. The same four scoring functions (ChemPLP, GS, CS, and ASP) were used. For example, with compound **12**, our docking results showed that the compounds bind to different part of the equivalent binding site (10 Å radius from residue Glu-375 for Hsp90αMD (PDB id: 3Q6M; x = −1.652, y = −64.237, z = 27.08) and residue Asp-367 for Hsp90β MD; Figure S1) on the two isoforms (Figure 2). The scale of the fitness scores derived from the docking algorithms showed that all eight compounds have higher scores and more hydrogen bonding interactions towards the binding pocket of Hsp90β MD when compared to the docking results to Hsp90α-MD (Table S7; Figure 3 and Figures S12–S18). For example, for compound **12**, the GS scoring function suggested that it does not have any hydrogen bonding interactions with the neighbouring amino acid residues of Hsp90α MD, but it forms three different hydrogen bonds with Hsp90β MD (Figure 3). The ligand binding positions that are generated by the search algorithm component of molecular docking showed that the most likely binding pose of each compound has the better fitness for the β-isoform than the α-isoform. These computational results correlate well with the experimental trend of *K*_D_ values for compounds **5**, **9**, **10**, **12**, **17,** and **24** from the binding assay. For compounds **8** and **22**, the differences could be due to inaccuracies in the predicted binding modes to the “real” configurations. Further studies, including competition binding experiments and protein X-ray crystallography, may give more information about the interactions of these two molecules to Hsp90 MD.

To check for the compatibility of the ligands with biological systems, their physicochemical properties were derived. The calculated molecular descriptors for the eight derivatives were molecular weight (MW), lipophilicity (logP), number of hydrogen bond donor (HD), number of hydrogen bond acceptor (HA), polar surface area (PSA), and number of rotatable bonds (RB), and are displayed in Table 2. For MW, the values span from lead-like, through drug-like space to known drug space (KDS; see Table 3 for definitions) [76,77,78]. HD for most compounds fit within the lead-like space [76,77]. The log P values lie in the range of 0.8 and 4.3 and the PSA values range from 91.2 to 153.9, with the latter reaching into KDS [78].

The Known Drug Indexes 2a and 2b (KDI_2a/2b_) [79] were also derived. The KDI reflect the overall balance of the molecular properties of ligands based on KDS. KDI_2a_ is additive with a maximum of 6.0 and for KDI_2b_ the indexes are multiplied giving 1.0 as its maximum. The average for KDI_2a_ for known drugs is 4.08 (± 1.27) and the ligands lie in the range of 3.63 to 5.68, i.e., they are well balanced compared to known drugs, except for ligand **17**, which has a large MW resulting in a low index. The KDI_2b_ gives a range of 0.02 to 0.70 with the average of know drugs being 0.18 (± 0.20), with ligands **12** and **22** with excellent values of 0.70. In general, the ligands are well balanced, with the notable exception of **17**, and can therefore be considered excellent starting points for further development.

## 3. Materials and Methods

### 3.1. Recombinant Production of Hsp90α/β MD

Plasmids pET-21a encoding Hsp90α/β MD were purchased from GenScript (Central, Hong Kong. For sequence, see Tables S3 and S4), which were then transformed into *Escherichia coli* BL21 (DE3) for protein production. In brief, cells were first grown in 2-YT media to an optical density at 600 nm wavelength (OD_600_) of around 0.6 to 0.8. Protein production was then induced by the addition of 0.2 mM isopropyl-1-thio-D-galactopyranoside (IPTG), which was then further incubated at 15 °C overnight. The harvested cells were resuspended in 25 mM Tris (pH 8.0) supplemented with Protease Inhibitor Cocktail (Abcam, Melbourne, Australia), 5 mM dithiothreitol (DTT), and 2 mM ethylenediaminetetraacetic acid (EDTA). The resuspended cells were lysed by sonication. The filtered cell lysate was applied to 1 mL HiTrap Q FF anion exchange chromatography column (GE Healthcare Life Sciences, Auckland, New Zealand) equilibrated with 25 mM Tris (pH 8.0), 2 mM EDTA, and 2 mM DTT. The protein was eluted with a linear gradient to 1 M NaCl from 0% to 100% over 70 min at a flow rate of 1 mL min^−1^. The protein was further purified by HiPrep 16/60 Sephacryl S-100 HR gel filtration column (GE Healthcare Life Sciences, Auckland, New Zealand) in 20 mM Tris (pH 7.5), 0.2 M KCl, 2 mM EDTA, and 2 mM DTT. The protein was then concentrated using Amicon Ultra-4 10K centrifugal filter (Merck Millipore, Auckland, New Zealand) and stored at −80 °C until use.

### 3.2. Molecular Docking

A blind docking of gambogic acid was conducted over the entire MD of Hsp90β and a binding pocket within a region consisted of residues 350 to 436 was identified. In order to validate the binding site as reported, gambogic acid was docked to the crystal structure of the MD of human Hsp90β (PDB id: 3PRY, resolution 2.28 Å) which was acquired from the Protein Data Bank (PDB) [80]. GS, CS, ChemPLP, and ASP scoring functions were used to validate the predicted binding modes and relative energies of the ligands using the GOLD v5.4 software suite. The Scigress Ultra v2.6 program (Version FJ 2.6 (EU 3.1.7), Fujitsu Limited, Wellington, New Zealand) was used to prepare the crystal structure as a docking scaffold, by adding hydrogen atoms and removing crystallographic water molecules. The basic amino acids lysine and arginine were defined as protonated. Furthermore, aspartic and glutamic acids were assumed deprotonated. The Scigress software suite was also used for structural optimization of the ligands via MM2 force field method [81]. Scores and root-mean-square deviation (RMSD) between the predicted ligand conformation of the scoring functions were compared to define the centre of binding pocket.

### 3.3. Pilot Screening

GOLD v5.4 software suite was used for virtual screening. Full experimental details were reported in Section 2.

### 3.4. Calculation of Molecular Descriptors

QikProp 3.2 software package (Schrödinger, New York, NY, USA) was used to calculate the molecular descriptors, which are the numerical values that assesses the properties of the compounds. The criterion of lead-like, drug-like, and KDS are listed in terms of the six respective molecular descriptors in Table 3 [76,77,78]. The reliability of QikProp is established for the calculated descriptors [82].

### 3.5. Intrinsic Tryptophan Fluorescence Spectroscopy

Fluorescence was measured using EnSpire Multimode Plate Reader (PerkinElmer, Melbourne, Australia). Experiments were performed in 20 µM of Hsp90 MD in 20 mM Tris (pH 7.5) and 0.2 M KCl. Different concentration of the virtual hits (InterBioScreen, Bar, Montenegro) or gambogic acid were added to the mixture. Excitation wavelength was 280 nm and intrinsic fluorescence was measured between 300 and 450 nm. The control experiment with Hsp90 MD on its own was conducted. Background fluorescence arising from the compound was subtracted to generate the final spectrum. The total volume per well of the 96-well microplate was 30 µL. Non-linear curve fitting of Equation (1) to the data was conducted using SigmaPlot 14.0 (Systat Software, San Jose, CA, USA). All tests were conducted in triplicate and the errors shown are standard deviation. *K*_D_ was calculated for each binder using Equation (1), where Δ_obs_ is the changes in fluorescence intensity from the titrations, Δ_max_ is the maximum fluorescence intensity change, [L_T_] represents the titrated ligand concentration, and [P_T_] indicates the protein concentration.

(1) $$\Delta_{\mathrm{obs}}=\Delta_{\max}\times \frac{\left(K_{D}+\left[L_{T}\right]+\left[P_{T}\right]\right)-\sqrt{{\left(K_{D}+\left[L_{T}\right]+\left[P_{T}\right]\right)}^{2}-\left(4\times \left[P_{T}\right]\times \left[L_{T}\right]\right)}}{2\times \left[P_{T}\right]}$$

## 4. Conclusions

Overall, by using an approach that combines both virtual screening and binding studies, we have successfully identified novel ligands that are selective for the β-isoform of Hsp90 MD. Molecular modelling was used to predict the most probable binding modes of the compounds to Hsp90 MD and intrinsic protein fluorescence was used to measure the binding affinity of our compounds to both isoforms of Hsp90 MD. Although the binding affinity and selectivity of the ligands for the β-isoform appears to be modest (low μM *K*_D_ values, ~5 to 7-fold for the best compounds), this is on par with a previous study that reported selective Hsp90β MD inhibitor (gambogic acid, low μM *K*_D_ value, and ~7-fold preference in terms of *K*_D_) [55]. In addition, in a recent study by Khandelwal et al., who reported selective inhibitors for Hsp90β NTD, their initial hits only showed 2–4-fold selectivity [62]. As all of our compounds fit into the known-drug space, drug-like space, or lead-like space areas, we believe our compounds are therefore good starting points for further developments of more selective Hsp90β MD inhibitors.

## Figures and Tables

**Table 1:** Binding affinity (*K*_D_) of our compounds to Hsp90α/β MD. Gambogic acid was used as a control.

Compound	*K*_D_ to Hsp90α MD/μM	*K*_D_ to Hsp90β MD/μM
**5**	470 ± 2	136 ± 1
**8**	284 ± 4	500
**9**	299 ± 3	57 ± 1
**10**	>500	164 ± 2
**12**	92 ± 0.8	<20
**17**	506 ± 4	285 ± 8
**22**	459 ± 7	>500
**24**	661 ± 3	265 ± 4
**Gambogic acid**	195 ± 5	33 ± 2

**Table 2:** Molecular descriptors of the hit compounds.

Compound	MW	HD	HA	Log P	PSA	RB	KDI_2a/b_
**5**	409.4	1.25	7	3.3	127.0	8	5.48/0.57
**8**	445.4	0.25	6.5	4.3	137.3	6	5.27/0.45
**9**	423.5	1.25	7	3.7	122.5	8	5.46/0.56
**10**	447.5	2	8.3	3.8	73.9	7	5.54/0.61
**12**	292.3	1	6.75	1.3	93.2	4	5.66/0.70
**17**	527.5	4	14.05	0.8	154.0	8	3.63/0.02
**22**	359.4	1.25	6	3.8	91.2	8	5.68/0.70
**24**	488.5	3	6.5	3.4	95.6	7	5.48/0.57

**Table 3:** Definition of lead-like, drug-like, and known drug space (KDS). The values given are the maximum value for each descriptor.

Molecular Descriptors	Lead-Like Space	Drug-Like Space	Known Drug Space
MW (g·mol^−1^)	300	500	800
Log *p*	3	5	6.5
HD	3	5	7
HA	3	10	15
PSA (Å^2^)	60	140	180
RB	3	10	17

## Supplementary Materials

Supplementary materials can be found at https://www.mdpi.com/1422-0067/20/21/5333/s1.

## Abbreviations

ASP	Astex Statistical Potential
ATP	Adenosine triphosphate
ChemPLP	Piecewise Linear Potential
CS	ChemScore
CTD	C-terminal domain
DTT	Dithiothreitol
EDTA	Ethylenediaminetetraacetic acid
GA	Genetic algorithm
GS	GoldScore
HA	Number of hydrogen bond acceptor
HD	Number of hydrogen bond donor
Hsp90	Heat shock protein 90
IPTG	Isopropyl-1-thio-D-galactopyranoside
*K*_D_	Dissociation constant
KDI	Known drug index
KDS	Known drug space
Log P	Lipophilicity
MD	Middle domain
MW	Molecular weight
NTD	N-terminal domain
OD_600_	Optical density at 600 nm wavelength
PDB	Protein data bank
PSA	Polar surface area
RB	Number of rotatable bonds
RMSD	Root-mean-square deviation
