# Supplementary material for: Identification of Isoform-Selective Ligands for the Middle Domain of Heat Shock Protein 90 (Hsp90)
Source: Int J Mol Sci. 2019 Oct 26;20(21):5333. doi: 10.3390/ijms20215333 (PMC6862331; doi:10.3390/ijms20215333)
Supplement: Supplementary file 1 [file ijms-20-05333-s001.pdf]

*Supplementary Information*

# Identification of Isoform-Selective Ligands for the Middle Domain of Heat Shock Protein 90 (Hsp90)


Oi Wei Mak [1], Raina Chand [1], Jóhannes Reynisson [1,2,*] and Ivanhoe K. H. Leung [1,3,*]

1 School of Chemical Sciences, The University of Auckland, Private Bag 92019, Victoria Street West, Auckland 1142, New Zealand.

2 School of Pharmacy and Bioengineering, Hornbeam Building, Keele University, Keele, Staffordshire ST5 5BG, United Kingdom.

3 Maurice Wilkins Centre for Molecular Biodiscovery, The University of Auckland, Private Bag 92019, Victoria Street West, Auckland 1142, New Zealand.

* Correspondence: j.reynisson@keele.ac.uk; Tel.: +44-1782-733-985 (J.R.); i.leung@auckland.ac.nz; Tel.: +64-9-923-1102 (I.K.H.L.)

```
Hsp90β    1   MPEEVHHG-----EEEVETFAFQAEIAQLMSLIINTFYSNKEIFLRELISNASDALDKIR  55
              MPEE         EEEVETFAFQAEIAQLMSLIINTFYSNKEIFLRELISN+SDALDKIR
Hsp90α    1   MPEETQTQDQPMEEEEVETFAFQAEIAQLMSLIINTFYSNKEIFLRELISNSSDALDKIR  60

Hsp90β   56   YESLTDPSKLDSGKELKIDIIPNPQERTLTLVDTGIGMTKADLINNLGTIAKSGTKAFME  115
              YESLTDPSKLDSGKEL I++IPN Q+RTLT+VDTGIGMTKADLINNLGTIAKSGTKAFME
Hsp90α   61   YESLTDPSKLDSGKELHINLIPNKQDRTLTIVDTGIGMTKADLINNLGTIAKSGTKAFME  120

Hsp90β  116   ALQAGADISMIGQFGVGFYSAYLVAEKVVVITKHNDDEQYAWESSAGGSFTVRADHGEPI  175
              ALQAGADISMIGQFGVGFYSAYLVAEKV VITKHNDDEQYAWESSAGGSFTVR D GEP+
Hsp90α  121   ALQAGADISMIGQFGVGFYSAYLVAEKVTVITKHNDDEQYAWESSAGGSFTVRTDTGEPM  180

Hsp90β  176   GRGTKVILHLKEDQTEYLEERRVKEVVKKHSQFIGYPITLYLEKEREKEISDDEAEEEKG  235
              GRGTKVILHLKEDQTEYLEERR+KE+VKKHSQFIGYPITL++EKER+KE+SDDEAEE++
Hsp90α  181   GRGTKVILHLKEDQTEYLEERRIKEIVKKHSQFIGYPITLFVEKERDKEVSDDEAEEKED  240

Hsp90β  236   EKEEEDKDDEEKPK---IEDVGSDEEDDSGKDKKKKTKKIKEKYIDQEELNKTKPIWTRN  292
              ++EE++K+++E      IEDVGSDEE++     KKK KKIKEKYIDQEELNKTKPIWTRN
Hsp90α  241   KEEEKEKEEKESEDKPEIEDVGSDEEEEKKDGDKKKKKKIKEKYIDQEELNKTKPIWTRN  300

Hsp90β  293   PDDITQEEYGEFYKSLTNDWEDHLAVKHFSVEGQLEFRALLFIPRRAPFDLFENKKKKNN  352
              PDDIT EEYGEFYKSLTNDWEDHLAVKHFSVEGQLEFRALLF+PRRAPFDLFEN+KKKNN
Hsp90α  301   PDDITNEEYGEFYKSLTNDWEDHLAVKHFSVEGQLEFRALLFVPRRAPFDLFENRKKKNN  360

Hsp90β  353   IKLYVRRVFIMDSCDELIPEYLNFIRGVVDSEDLPLNISREMLQQSKILKVIRKNIVKKC  412
              IKLYVRRVFIMD+C+ELIPEYLNFIRGVVDSEDLPLNISREMLQQSKILKVIRKN+VKKC
Hsp90α  361   IKLYVRRVFIMDNCEELIPEYLNFIRGVVDSEDLPLNISREMLQQSKILKVIRKNLVKKC  420

Hsp90β  413   LELFSELAEDKENYKKFYEAFSKNLKLGIHEDSTNRRRLSELLRYHTSQSGDEMTSLSEY  472
              LELF+ELAEDKENYKKFYE FSKN+KLGIHEDS NR++LSELLRY+TS SGDEM SL +Y
Hsp90α  421   LELFTELAEDKENYKKFYEQFSKNIKLGIHEDSQNRKKLSELLRYYTSASGDEMVSLKDY  480

Hsp90β  473   VSRMKETQKSIYYITGESKEQVANSAFVERVRKRGFEVVYMTEPIDEYCVQQLKEFDGKS  532
              +RMKE QK IYYITGE+K+QVANSAFVER+RK G EV+YM EPIDEYCVQQLKEF+GK+
Hsp90α  481   CTRMKENQKHIYYITGETKDQVANSAFVERLRKHGLEVIYMIEPIDEYCVQQLKEFEGKT  540

Hsp90β  533   LVSVTKEGLELPEDEEEKKKMEESKAKFENLCKLMKEILDKKVEKVTISNRLVSSPCCIV  592
              LVSVTKEGLELPEDEEEKKK EE K KFENLCK+MK+IL+KKVEKV +SNRLV+SPCCIV
Hsp90α  541   LVSVTKEGLELPEDEEEKKKQEEKKTKFENLCKIMKDILEKKVEKVVVSNRLVTSPCCIV  600

Hsp90β  593   TSTYGWTANMERIMKAQALRDNSTMGYMMAKKHLEINPDHPIVETLRQKAEADKNDKAVK  652
              TSTYGWTANMERIMKAQALRDNSTMGYM AKKHLEINPDH I+ETLRQKAEADKNDK+VK
Hsp90α  601   TSTYGWTANMERIMKAQALRDNSTMGYMAAKKHLEINPDHSIIETLRQKAEADKNDKSVK  660

Hsp90β  653   DLVVLLFETALLSSGFSLEDPQTHSNRIYRMIKLGLGIDEDEVAAEEPNAAVPDEIPPLE  712
              DLV+LL+ETALLSSGFSLEDPQTH+NRIYRMIKLGLGIDED+  A++ +AAV +E+PPLE
Hsp90α  661   DLVILLYETALLSSGFSLEDPQTHANRIYRMIKLGLGIDEDDPTADDTSAAVTEEMPPLE  720

Hsp90β  713   GDEDASRMEEVD  724
              GD+D SRMEEVD
Hsp90α  721   GDDDTSRMEEVD  732
```

Supplementary Table S1. Sequence alignment of human Hsp90α (https://www.uniprot.org/uniprot/P07900; Middle domain is highlighted in green) and Hsp90β (https://www.uniprot.org/uniprot/P08238; Middle domain is highlighted in yellow). Sequence alignment was conducted using the Protein BLAST tool (Basic Local Alignment Search Tool; https://blast.ncbi.nlm.nih.gov/Blast.cgi)

| Amino acid residues | ChemPLP | GoldScore | RMSD (Å) | ChemScore | RMSD (Å) | ASP | RMSD (Å) |
|---|---|---|---|---|---|---|---|
| Ile-353 | 50.0 | 56.3 | 8.3 | 18.6 | 2.9 | 18.8 | 10.2 |
| Ser-365 | 62.9 | 49.4 | 8.4 | 20.3 | 1.1 | 24.0 | 6.9 |
| Asp-367 | 69.0 | 48.7 | 7.3 | 20.7 | 1.1 | 22.2 | 0.9 |
| Ile-370 | 74.3 | 50.7 | 10.3 | 21.5 | 7.0 | 26.3 | 1.7 |
| Glu-372 | 54.4 | 53.4 | 6.5 | 21.6 | 5.3 | 22.9 | 8.5 |
| Asn-436 | 62.3 | 51.3 | 3.8 | 29.3 | 2.1 | 26.6 | 6.9 |

Supplementary Table S2. Scoring functions for the docking of gambogic acid on respective six potential binding sites of Hsp90β-MD (PDB ID: 3PRY). Root Mean Square Deviation (RMSD) calculations from each scoring function were compared by using ChemPLP as the reference for each docking study, in order to determine the consistency of the ligand poses within the active site. Note: A total of 87 binding site were spotted and defined for molecular docking. Only six out of 87 residues (350-436) are active and dockable while the rest of the spots have inadequate genetic algorithm rates. This might be due to the deficiency of donors and acceptors nor the solvent accessible atoms within the active sites.

| Derivative | ASP | ChemScore | GoldScore | ChemPLP |
|---|---|---|---|---|
| Gambogic acid | 22.2 | 20.7 | 48.7 | 69.0 |
| **1** | 26.5 | 23.5 | 67.1 | 67.5 |
| **2** | 36.4 | 27.6 | 76.6 | 78.2 |
| **3** | 37.8 | 21.9 | 68.3 | 78.7 |
| **4** | 30.0 | 36.7 | 65.4 | 76.2 |
| **5** | 26.4 | 22.9 | 68.4 | 73.4 |
| **6** | 29.6 | 24.9 | 62.0 | 76.3 |
| **7** | 34.0 | 29.9 | 77.4 | 86.5 |
| **8** | 36.9 | 25.5 | 78.7 | 79.9 |
| **9** | 27.5 | 21.2 | 66.0 | 77.8 |
| **10** | 29.7 | 24.8 | 66.1 | 63.1 |
| **11** | 27.5 | 24.9 | 60.2 | 63.7 |
| **12** | 27.4 | 24.7 | 60.3 | 60.4 |
| **13** | 30.1 | 22.7 | 64.3 | 71.7 |
| **14** | 25.3 | 20.6 | 60.5 | 63.1 |
| **15** | 27.3 | 21.2 | 62.7 | 71.4 |
| **16** | 26.5 | 20.5 | 67.9 | 68.2 |
| **17** | 30.4 | 23.2 | 61.0 | 73.0 |
| **18** | 30.6 | 26.7 | 73.8 | 81.7 |
| **19** | 31.0 | 27.9 | 61.2 | 75.8 |
| **20** | 26.2 | 22.5 | 63.5 | 65.1 |
| **21** | 26.6 | 22.7 | 62.3 | 75.7 |
| **22** | 26.8 | 21.7 | 64.5 | 70.8 |
| **23** | 31.0 | 22.8 | 64.2 | 75.0 |
| **24** | 32.8 | 30.3 | 74.3 | 79.4 |

Supplementary Table S3. Results of the scoring functions for the docking of gambogic acid and the 24 selected virtual hits against Hsp90β MD.

```
        10         20         30         40         50
MPEETQTQDQ PMEEEEVETF AFQAEIAQLM SLIINTFYSN KEIFLRELIS
        60         70         80         90        100
NSSDALDKIR YESLTDPSKL DSGKELHINL IPNKQDRTLT IVDTGIGMTK
       110        120        130        140        150
ADLINNLGTI AKSGTKAFME ALQAGADISM IGQFGVGFYS AYLVAEKVTV
       160        170        180        190        200
ITKHNDDEQY AWESSAGGSF TVRTDTGEPM GRGTKVILHL KEDQTEYLEE
       210        220        230        240        250
RRIKEIVKKH SQFIGYPITL FVEKERDKEV SDDEAEEKED KEEEKEKEEK
       260        270        280        290        300
ESEDKPEIED VGSDEEEEKK DGDKKKKKKI KEKYIDQEEL NKTKPIWTRN
       310        320        330        340        350
PDDITNEEYG EFYKSLTNDW EDHLAVKHFS VEGQLEFRAL LFVPRRAPFD
       360        370        380        390        400
LFENRKKKNN IKLYVRRVFI MDNCEELIPE YLNFIRGVVD SEDLPLNISR
       410        420        430        440        450
EMLQQSKILK VIRKNLVKKC LELFTELAED KENYKKFYEQ FSKNIKLGIH
       460        470        480        490        500
EDSQNRKKLS ELLRYYTSAS GDEMVSLKDY CTRMKENQKH IYYITGETKD
       510        520        530        540        550
QVANSAFVER LRKHGLEVIY MIEPIDEYCV QQLKEFEGKT LVSVTKEGLE
       560        570        580        590        600
LPEDEEEKKK QEEKKTKFEN LCKIMKDILE KKVEKVVVSN RLVTSPCCIV
       610        620        630        640        650
TSTYGWTANM ERIMKAQALR DNSTMGYMAA KKHLEINPDH SIIETLRQKA
       660        670        680        690        700
EADKNDKSVK DLVILLYETA LLSSGFSLED PQTHANRIYR MIKLGLGIDE
       710        720        730
DDPTADDTSA AVTEEMPPLE GDDDTSRMEE VD
```

Supplementary Table S4. Sequence of human Hsp90α. Red indicates the sequence of the middle domain (residues 286-546) that was used in this study.

```
        10         20         30         40         50
MPEEVHHGEE EVETFAFQAE IAQLMSLIIN TFYSNKEIFL RELISNASDA
        60         70         80         90        100
LDKIRYESLT DPSKLDSGKE LKIDIIPNPQ ERTLTLVDTG IGMTKADLIN
       110        120        130        140        150
NLGTIAKSGT KAFMEALQAG ADISMIGQFG VGFYSAYLVA EKVVVITKHN
       160        170        180        190        200
DDEQYAWESS AGGSFTVRAD HGEPIGRGTK VILHLKEDQT EYLEERRVKE
       210        220        230        240        250
VVKKHSQFIG YPITLYLEKE REKEISDDEA EEEKGEKEEE DKDDEEKPKI
       260        270        280        290        300
EDVGSDEEDD SGKDKKKKTK KIKEKYIDQE ELNKTKPIWT RNPDDITQEE
       310        320        330        340        350
YGEFYKSLTN DWEDHLAVKH FSVEGQLEFR ALLFIPRRAP FDLFENKKKK
       360        370        380        390        400
NNIKLYVRRV FIMDSCDELI PEYLNFIRGV VDSEDLPLNI SREMLQQSKI
       410        420        430        440        450
LKVIRKNIVK KCLELFSELA EDKENYKKFY EAFSKNLKLG IHEDSTNRRR
       460        470        480        490        500
LSELLRYHTS QSGDEMTSLS EYVSRMKETQ KSIYYITGES KEQVANSAFV
       510        520        530        540        550
ERVRKRGFEV VYMTEPIDEY CVQQLKEFDG KSLVSVTKEG LELPEDEEEK
       560        570        580        590        600
KKMEESKAKF ENLCKLMKEI LDKKVEKVTI SNRLVSSPCC IVTSTYGWTA
       610        620        630        640        650
NMERIMKAQA LRDNSTMGYM MAKKHLEINP DHPIVETLRQ KAEADKNDKA
       660        670        680        690        700
VKDLVVLLFE TALLSSGFSL EDPQTHSNRI YRMIKLGLGI DEDEVAAEEP
       710        720
NAAVPDEIPP LEGDEDASRM EEVD
```

Supplementary Table S5. Sequence of human Hsp90β. Red indicates the sequence of the middle domain (residues 294-554) that was used in this study.

| Compound | Formula | Name |
|---|---|---|
| **5** | $C_{23}H_{23}NO_6$ | (2*R*)-2-(2-((2-oxo-4-phenyl-2*H*-chromen-7-yl)oxy)propanamido)pentanoic acid |
| **8** | $C_{25}H_{19}NO_7$ | 5-hydroxy-4-oxo-2-phenyl-4*H*-chromen-7-yl ((benzyloxy)carbonyl)glycinate |
| **9** | $C_{24}H_{25}NO_6$ | (2*R*)-3-methyl-2-(2-((2-oxo-4-phenyl-2*H*-chromen-7-yl)oxy)propanamido)pentanoic acid |
| **10** | $C_{26}H_{29}N_3O_4$ | (*S*)-1,2,3-trimethoxy-7-(methylamino)-10-((pyridin-3-ylmethyl)amino)-6,7-dihydrobenzo[a]heptalen-9(5*H*)-one |
| **12** | $C_{18}H_{12}O_4$ | 2-hydroxy-3-(2-oxo-2-phenylethyl)naphthalene-1,4-dione |
| **17** | $C_{27}H_{29}NO_{10}$ | *N*-((2*S*,3*R*,4*R*,5*S*,6*R*)-2-((3-(3,4-dihydro-2*H*-benzo[*b*][1,4]dioxepin-7-yl)-2-methyl-4-oxo-4*H*-chromen-7-yl)oxy)-4,5-dihydroxy-6-(hydroxymethyl)tetrahydro-2*H*-pyran-3-yl)acetamide |
| **22** | $C_{19}H_{21}NO_6$ | (3,4,5-trimethoxybenzoyl)phenylalanine |
| **24** | $C_{28}H_{28}N_2O_6$ | 6-(benzyloxy)-1-(3,4,5-trimethoxyphenyl)-2,3,4,9-tetrahydro-1*H*-pyrido[3,4-b]indole-3-carboxylic acid |

Supplementary Table S6. Compound number, formula and chemical name of the hits that we obtained from the binding studies.

| Ligand | Docking at $\alpha$-isoform | | | | Docking at β-isoform | | | |
|---|---|---|---|---|---|---|---|---|
| | ChemPLP | CS | GS | ASP | ChemPLP | CS | GS | ASP |
| **5** | 45.6 | 14.7 | 39.1 | 20.8 | 73.4 | 22.9 | 68.4 | 26.4 |
| **8** | 55.1 | 16.8 | 50.3 | 25.6 | 79.9 | 25.5 | 78.7 | 36.9 |
| **9** | 54.8 | 16.6 | 40.1 | 22.1 | 77.8 | 21.2 | 66.0 | 27.5 |
| **10** | 42.3 | 14.6 | 38.4 | 42.4 | 63.1 | 24.8 | 66.1 | 29.7 |
| **12** | 42.0 | 16.8 | 39.4 | 17.3 | 60.4 | 24.7 | 60.3 | 27.4 |
| **17** | 47.1 | 8.7 | 47.9 | 22.3 | 73.0 | 23.2 | 61.0 | 30.4 |
| **22** | 48.3 | 12.4 | 46.9 | 19.8 | 70.8 | 21.7 | 64.5 | 26.8 |
| **24** | 45.8 | 15.7 | 50.9 | 22.6 | 79.4 | 30.3 | 74.3 | 32.8 |

Supplementary Table S7. Docking scores from the binders at the defined binding site of Hsp90α MD and Hsp90β MD respectively.

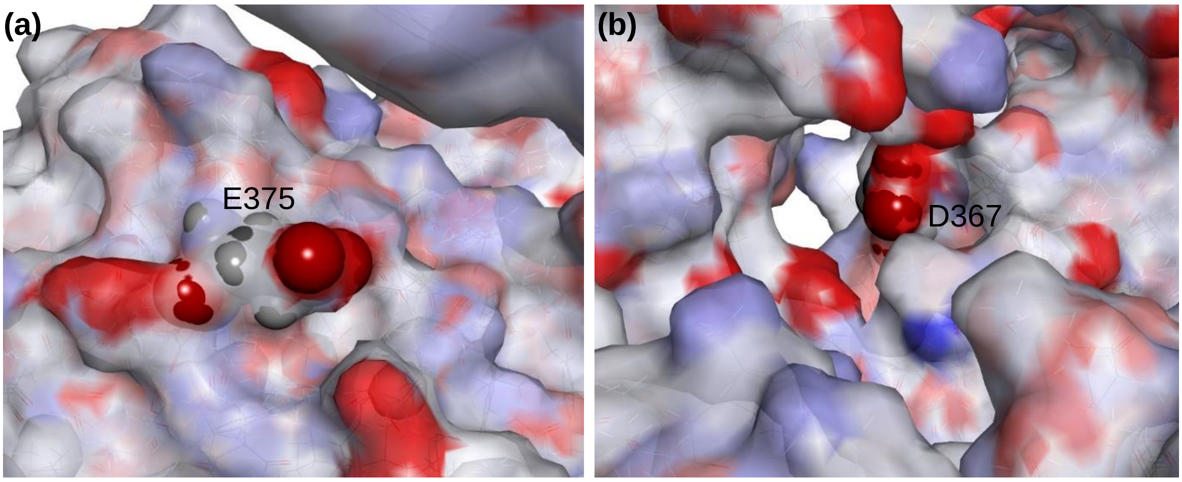


Supplementary Figure S1. Surface electrostatic potential map of (a) the small molecule binding site of Hsp90α (hot spot at residue E375). The binding site is defined as 10 Å radius from residue E375 for Hsp90αMD (PDB id: 3Q6M; x= -1.652, y= -64.237, z= 27.08), and (b) the small molecule binding site of Hsp90β (hot spot at residue D367). The binding site is defined as 10 Å radius from D367 (PDB ID: 3PRY; x = 8.806, y = 23.993, z = 27.785). Red depicts a negative partial charge on the surface, blue depicts positive partial charge and grey shows neutral/lipophilic regions. The hot spot residues were displayed as CPK space-filling models.

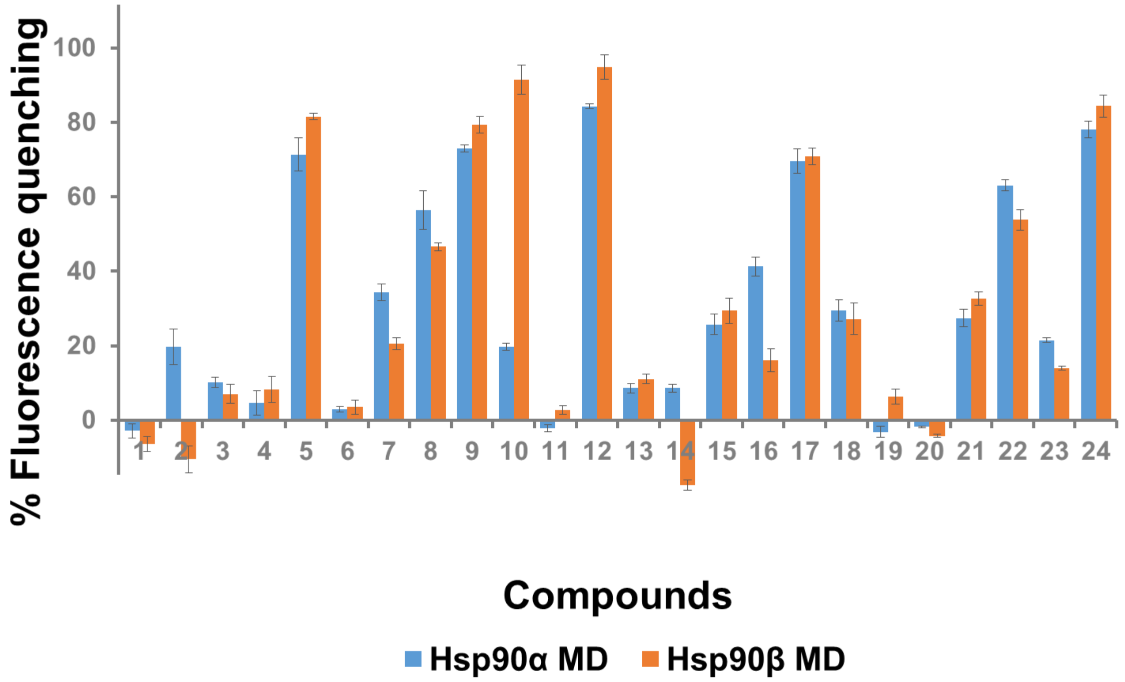


Supplementary Figure S2. Screening of the virtual hits (1 mM) to Hsp90α/β MD (20 μM) by intrinsic protein fluorescence. Percentage fluorescence quenching was calculated with the equation below.

$$\%\ Fluorescence\ quenching = \frac{(I_{protein} - I_{protein+compound})}{I_{protein}} \times 100\%$$

In which $I_{protein}$ denotes intrinsic fluorescence intensity of the protein in the absence of any compound, $I_{protein+compound}$ denotes intrinsic fluorescence intensity of the protein in the presence of the compound. Experiments were conducted in triplicate. Errors shown are standard derivation.

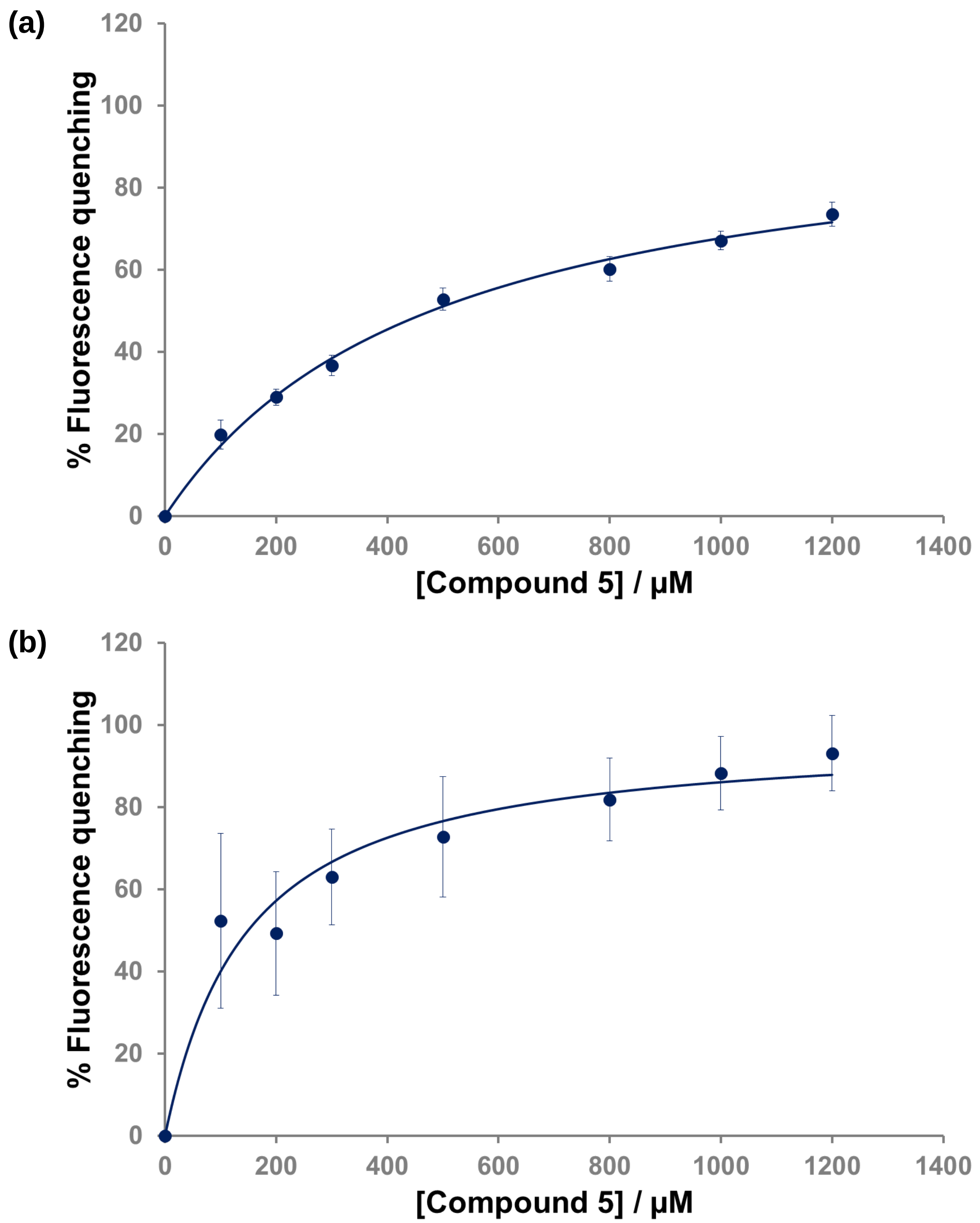


Supplementary Figure S3. $K_D$ determination by intrinsic protein fluorescence spectroscopy. (a) Titration of compound **5** to Hsp90α MD; (b) Titration of compound **5** to Hsp90β MD. Experiments were conducted in triplicate. Errors shown are standard derivation.

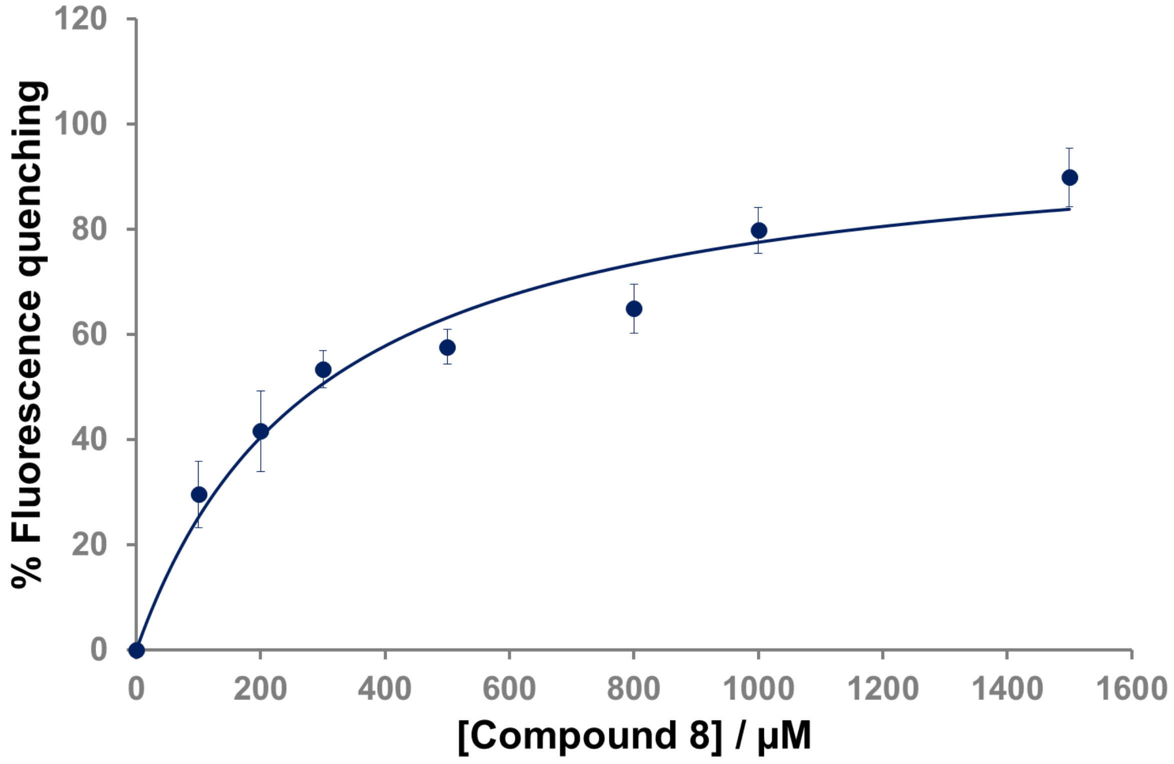


Supplementary Figure S4. $K_D$ determination by intrinsic protein fluorescence spectroscopy. Titration of compound **8** to Hsp90α MD.

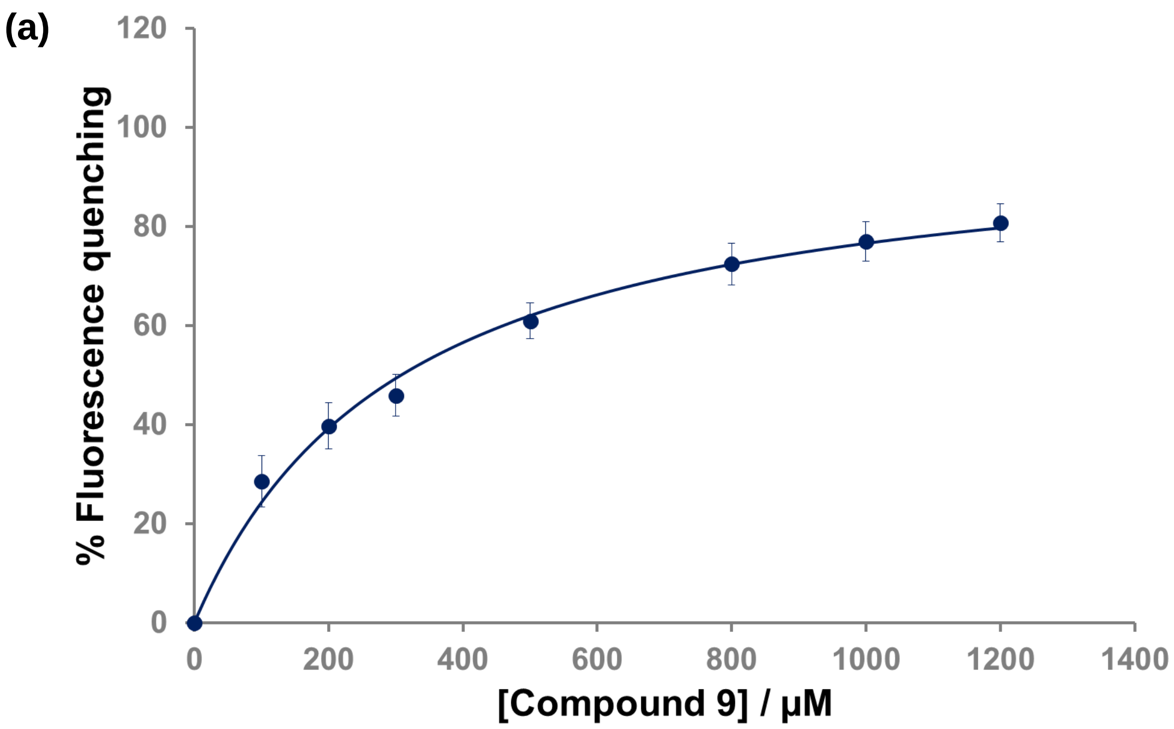


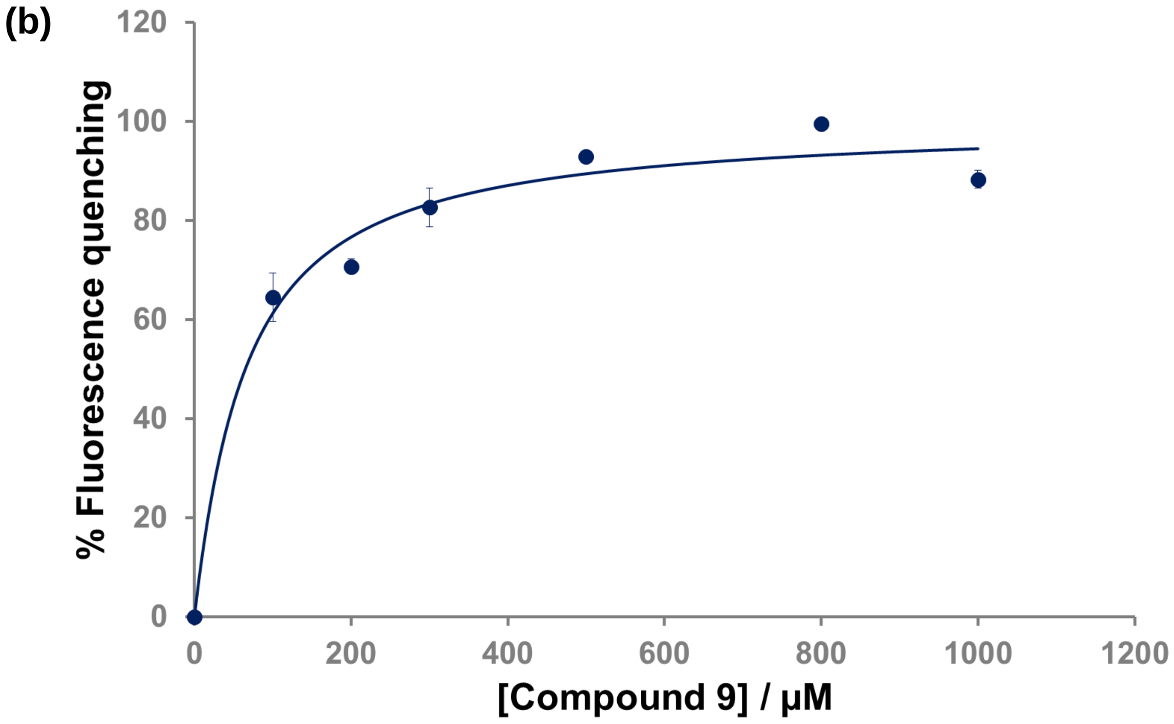


Supplementary Figure S5. $K_D$ determination by intrinsic protein fluorescence spectroscopy. (a) Titration of compound **9** to Hsp90α MD; (b) Titration of compound **9** to Hsp90β MD. Experiments were conducted in triplicate. Errors shown are standard derivation.

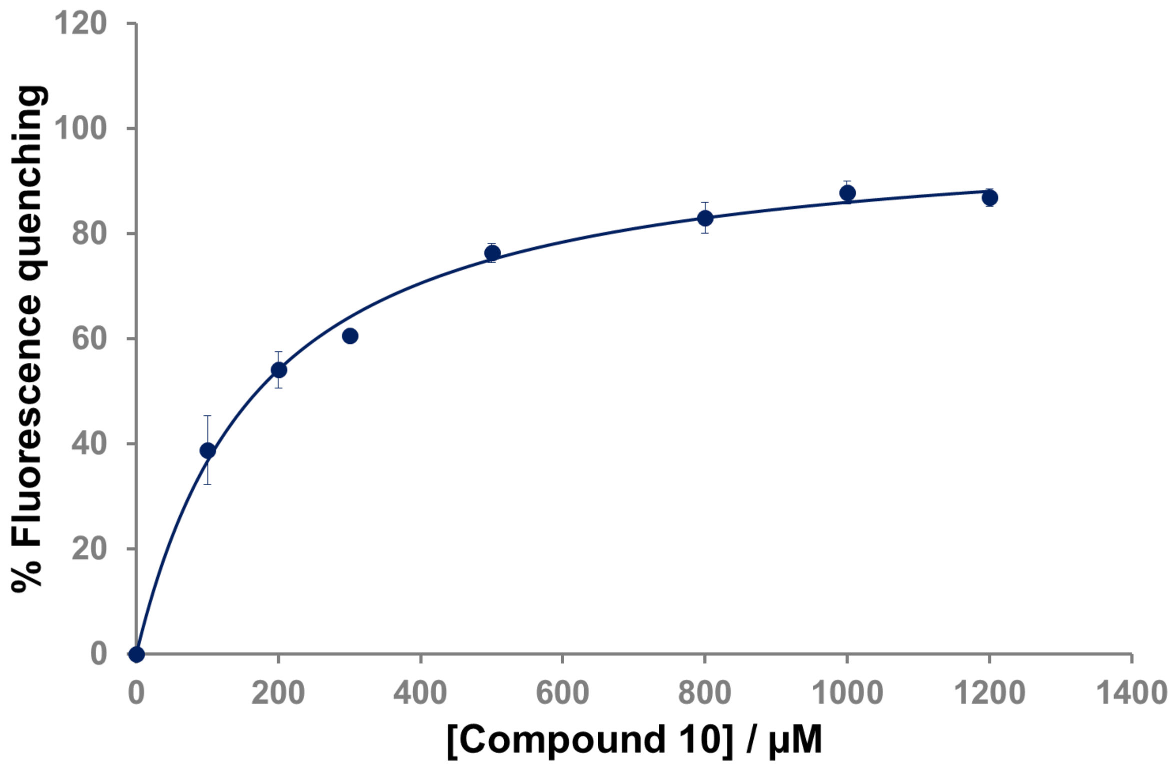


Supplementary Figure S6. $K_D$ determination by intrinsic protein fluorescence spectroscopy. Titration of compound **10** to Hsp90β MD.

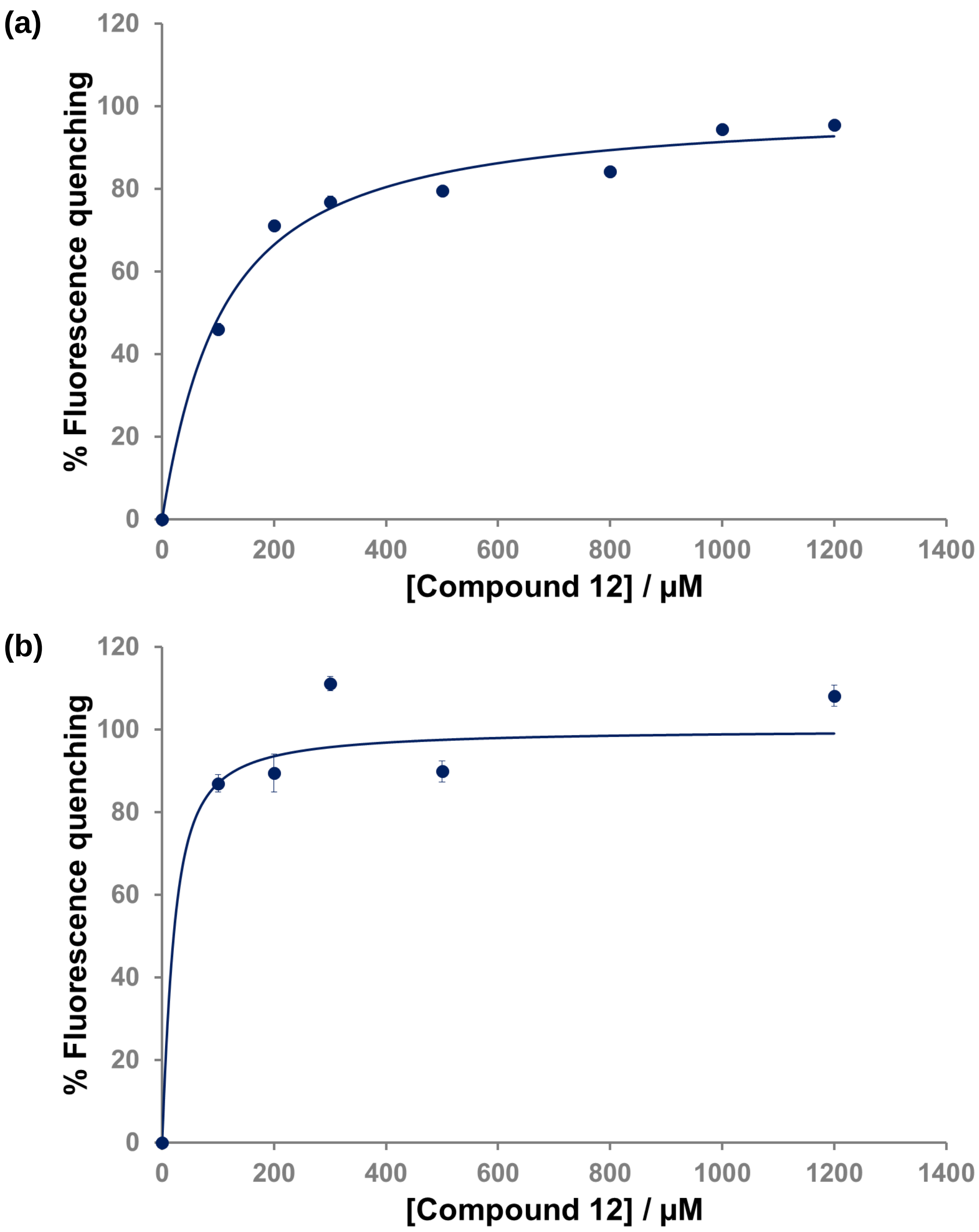


Supplementary Figure S7. $K_D$ determination by intrinsic protein fluorescence spectroscopy. (a) Titration of compound **12** to Hsp90α MD; (b) Titration of compound **12** to Hsp90β MD. Experiments were conducted in triplicate. Errors shown are standard derivation.

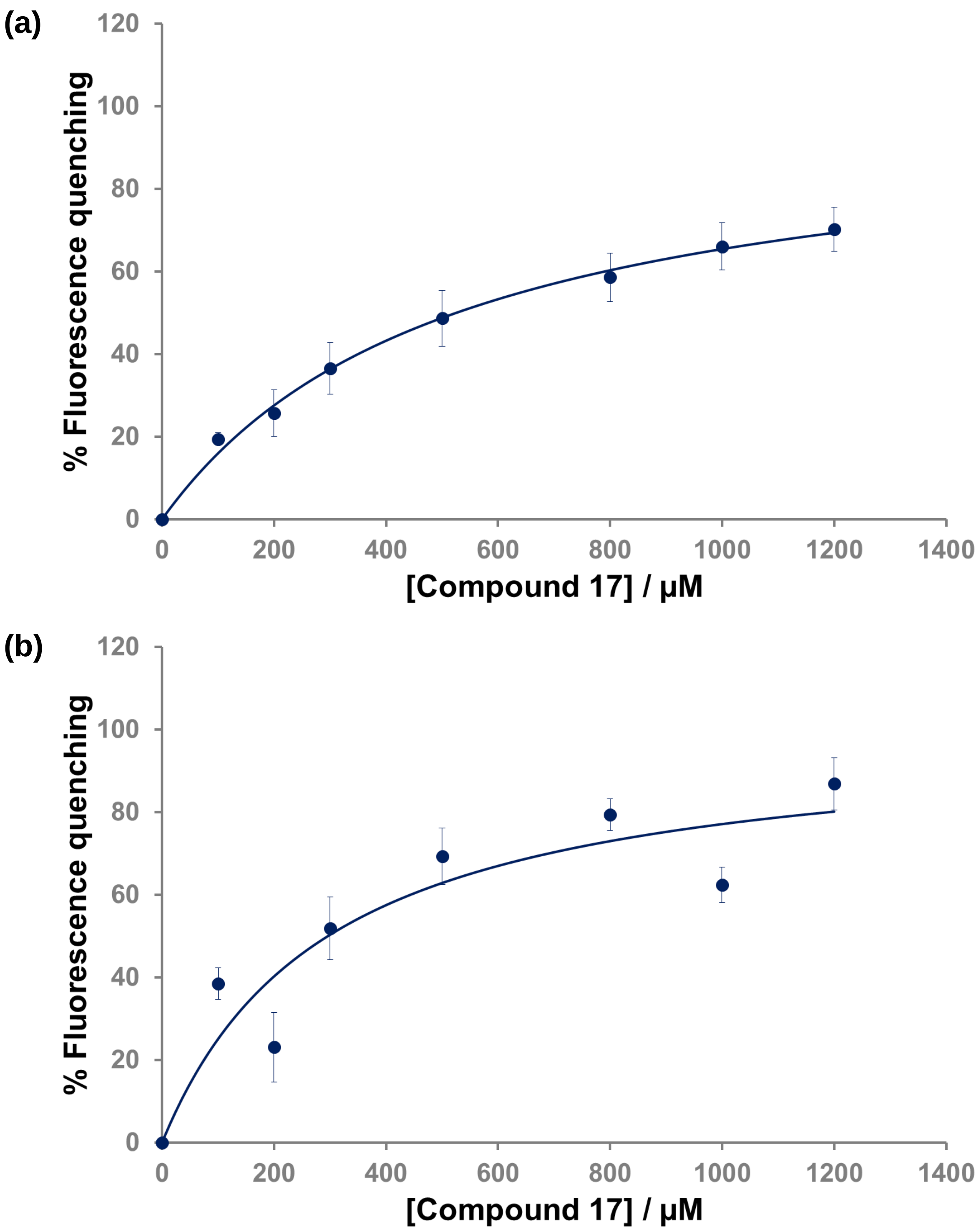


Supplementary Figure S8. $K_D$ determination by intrinsic protein fluorescence spectroscopy. (a) Titration of compound **17** to Hsp90α MD; (b) Titration of compound **17** to Hsp90β MD. Experiments were conducted in triplicate. Errors shown are standard derivation.

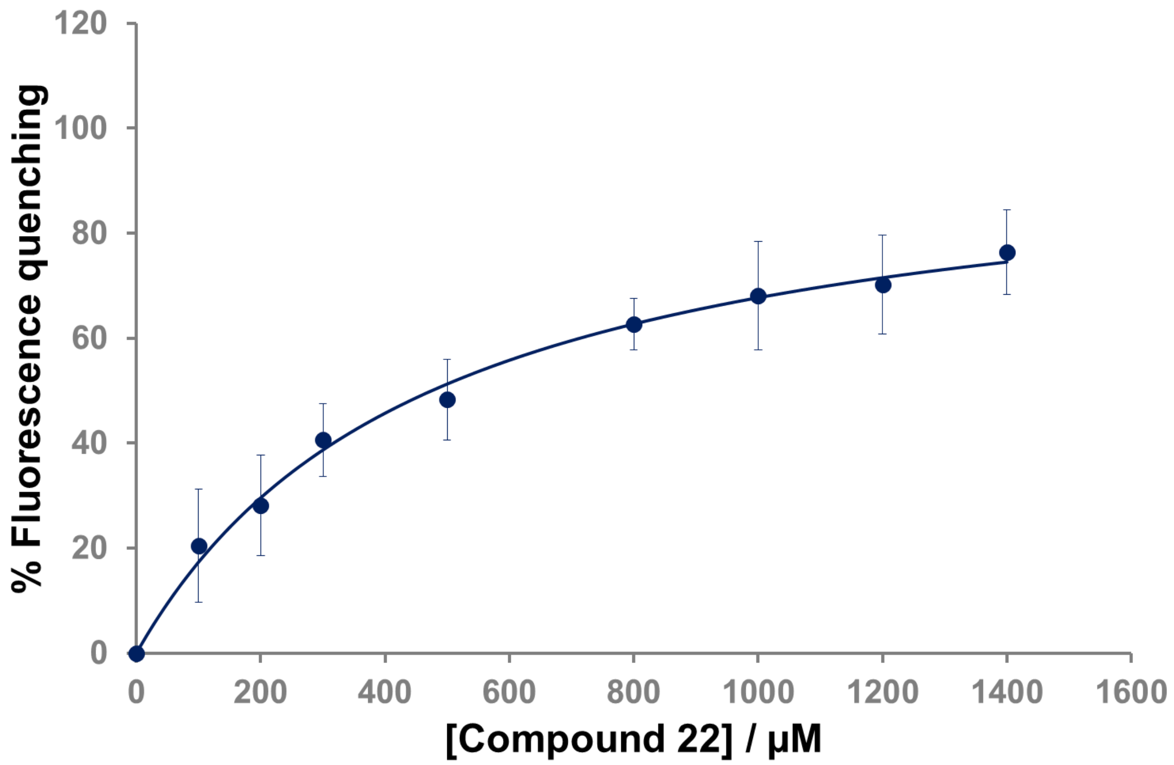


Supplementary Figure S9. $K_D$ determination by intrinsic protein fluorescence spectroscopy. Titration of compound **22** to Hsp90α MD.

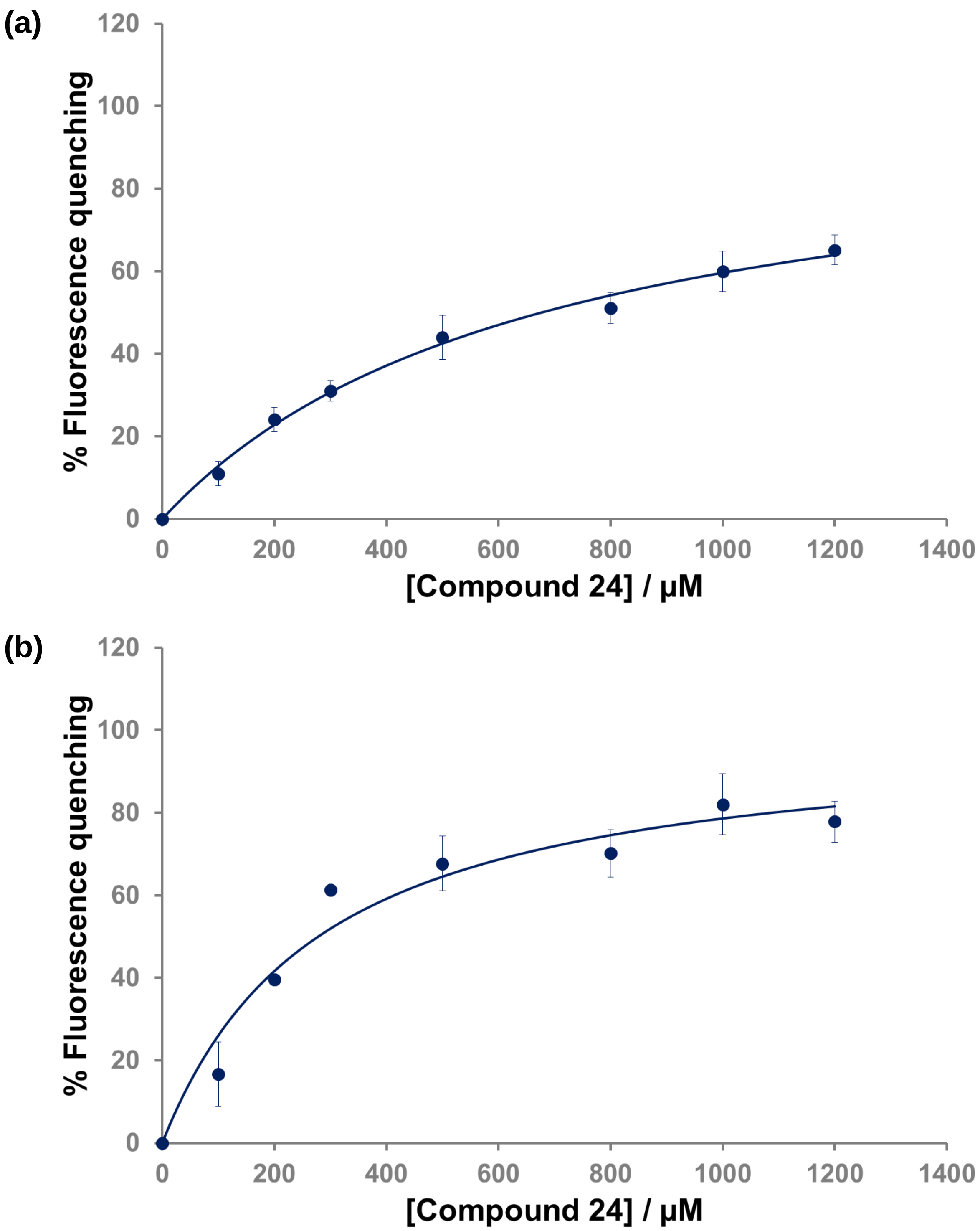


Supplementary Figure S10. $K_D$ determination by intrinsic protein fluorescence spectroscopy. (a) Titration of compound **24** to Hsp90α MD; (b) Titration of compound **24** to Hsp90β MD. Experiments were conducted in triplicate. Errors shown are standard derivation.

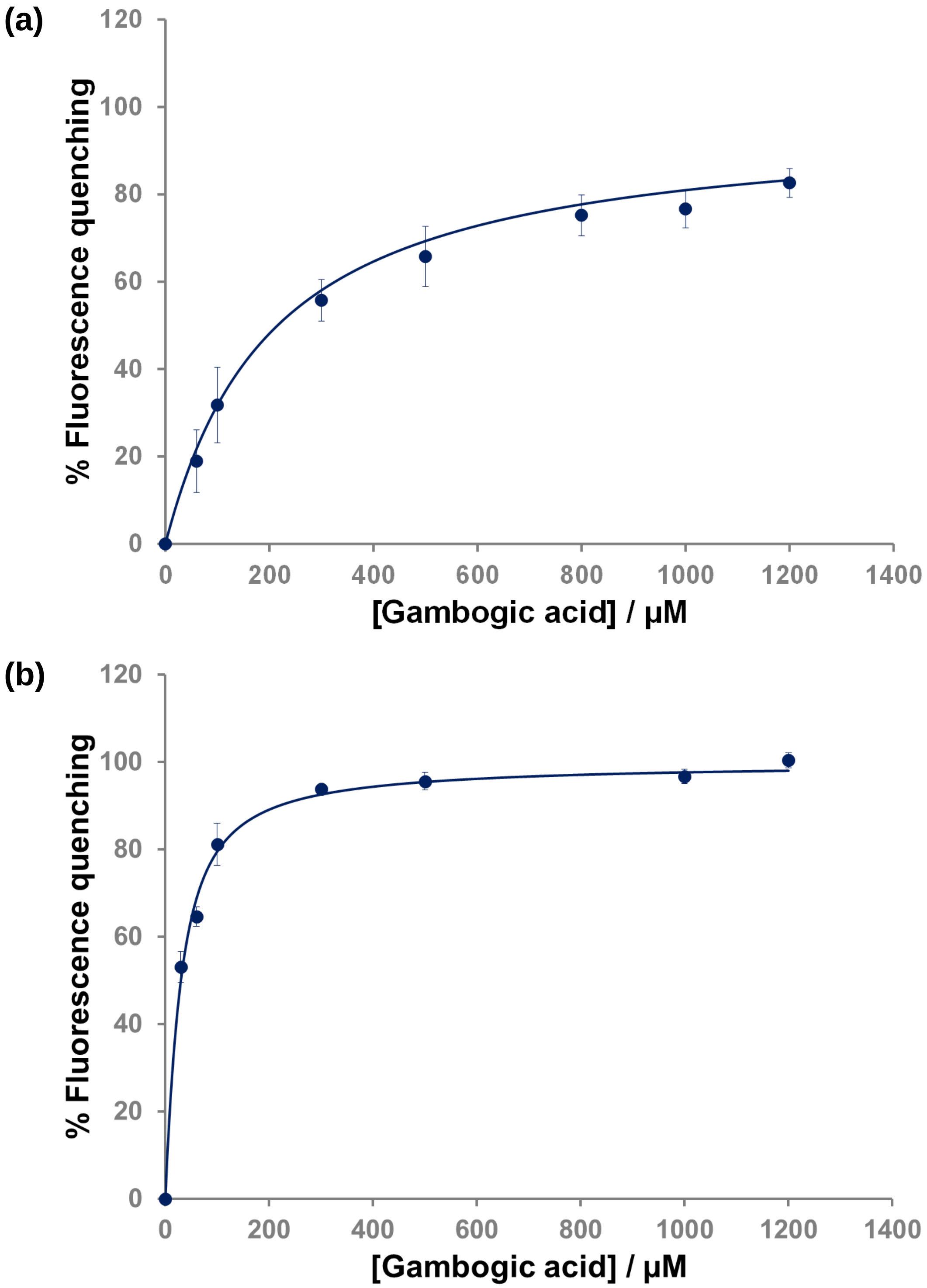


Supplementary Figure S11. $K_D$ determination by intrinsic protein fluorescence spectroscopy. (a) Titration of gambogic acid to Hsp90α MD; (b) Titration of gambogic acid to Hsp90β MD. Experiments were conducted in triplicate. Errors shown are standard derivation.

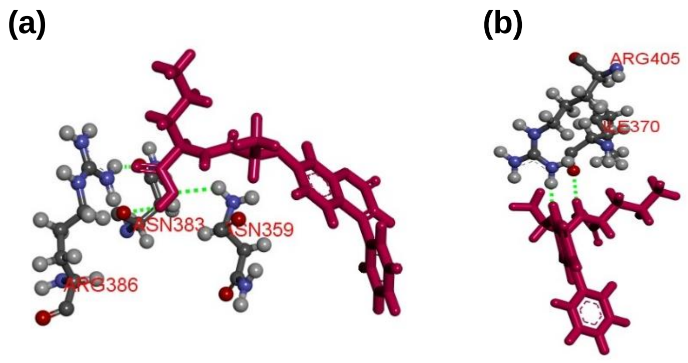


Supplementary Figure S12. Predicted binding modes and interactions of compound **5** to the Hsp90 isoforms. (a) Hydrogen bond interactions (depicted as green dotted lines) between compound **5** and Asn-359, Asn-383 and Arg-386 of Hsp90α; (b) Hydrogen bond interactions between compound **5** and Ile-370 and Arg-405 of Hsp90β. Both of the displays were processed from the ligand poses as predicted by the GS scoring function.

(a) (b)

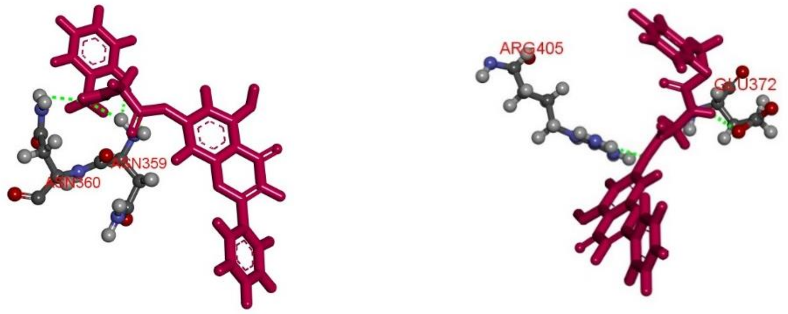


Supplementary Figure S13. Predicted binding modes and interactions of compound **8** to the Hsp90 isoforms. (a) Hydrogen bond interactions (depicted as green dotted lines) between compound **8** and Asn-359 and Arg-360 of Hsp90α; (b) Hydrogen bond interactions between compound **8** and Ile-370 and Arg-405 of Hsp90β. Both of the displays were processed from the ligand poses as predicted by the GS scoring function.

(a) (b)

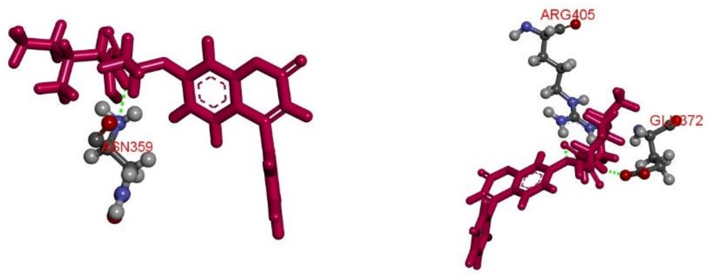


Supplementary Figure S14. Predicted binding modes and interactions of compound **9** to the Hsp90 isoforms. (a) Hydrogen bond interactions (depicted as green dotted lines) between compound **9** and Asn-359 of Hsp90α; (b) Hydrogen bond interactions between compound **9** and Glu-372 and Arg-405 of Hsp90β. Both of the displays were processed from the ligand poses as predicted by the GS scoring function.

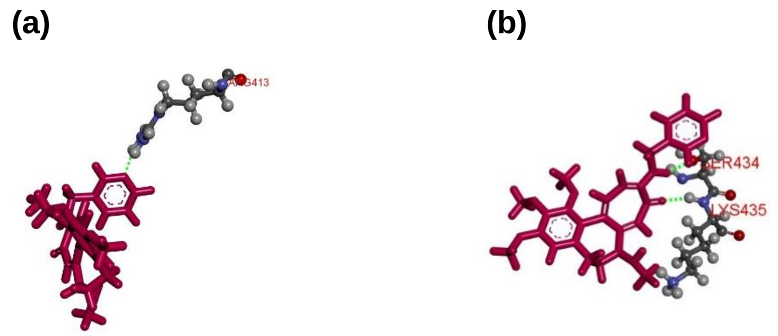


Supplementary Figure S15. Predicted binding modes and interactions of compound **10** to the Hsp90 isoforms. (a) Hydrogen bond interactions (depicted as green dotted lines) between compound **10** and Arg-413 of Hsp90α; (b) Hydrogen bond interactions between compound **10** and Ser-343 and Lys-435 of Hsp90β. Both of the displays were processed from the ligand poses as predicted by the GS scoring function.

(a) (b)

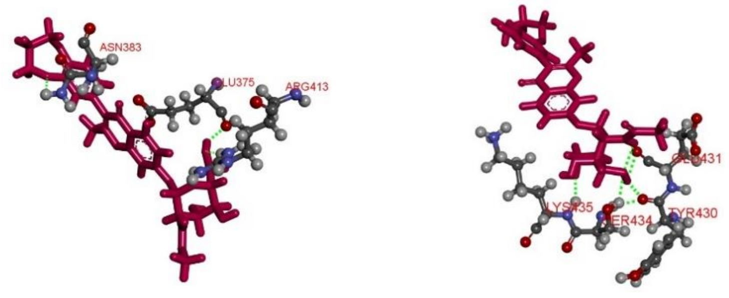


Supplementary Figure S16. Predicted binding modes and interactions of compound **18** to the Hsp90 isoforms. (a) Hydrogen bond interactions (depicted as green dotted lines) between compound **18** and Glu-375, Asn-383 and Arg-413 of Hsp90α; (b) Hydrogen bond interactions between compound **18** and Tyr-430, Glu-431, Ser-434 and Lys-435 of Hsp90β. Both of the displays were processed from the ligand poses as predicted by the GS scoring function.

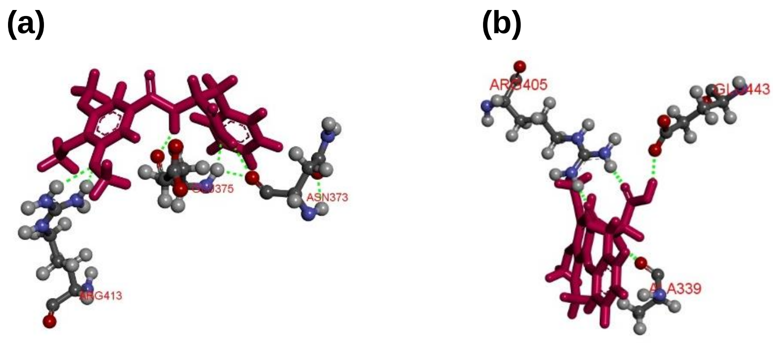


Supplementary Figure S17. Predicted binding modes and interactions of compound **22** to the Hsp90 isoforms. (a) Hydrogen bond interactions (depicted as green dotted lines) between compound **22** and Glu-375, Asn-373 and Arg-413 of Hsp90α; (b) Hydrogen bond interactions between compound **22** and Ala-339, Arg-405 and Glu-443 of Hsp90β. Both of the displays were processed from the ligand poses as predicted by the GS scoring function.

(a) (b)

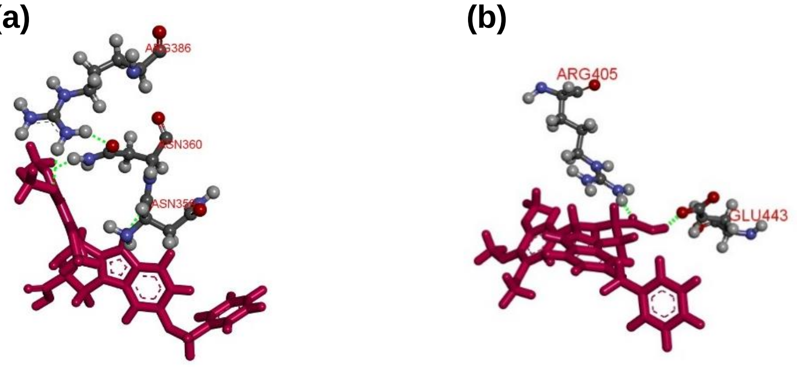


Supplementary Figure S18. Predicted binding modes and interactions of compound **24** to the Hsp90 isoforms. (a) Hydrogen bond interactions (depicted as green dotted lines) between compound **24** and Asn-359, Asn-360 and Arg-386 of Hsp90α; (b) Hydrogen bond interactions between compound **24** and Arg-405 and Glu-443 of Hsp90β. Both of the displays were processed from the ligand poses as predicted by the GS scoring function.
